# The effects of dietary cottonseed meal and oil supplementation on laying performance and egg quality of laying hens

**DOI:** 10.1002/fsn3.1112

**Published:** 2019-06-14

**Authors:** Yang Mu, Luo‐Yi Zhu, Ao Yang, Xin Gao, Niya Zhang, Lvhui Sun, Desheng Qi

**Affiliations:** ^1^ Department of Animal Nutrition and Feed Science, College of Animal Science and Technology Huazhong Agricultural University Wuhan China; ^2^ School of food and biological engineering Hubei University of Technology Wuhan China

**Keywords:** cottonseed meal, cottonseed oil, egg quality, laying hen, laying performance

## Abstract

Cottonseed meal (CSM) and cottonseed oil (CSO), two cottonseed products, are rich in protein and lipids, respectively, but their use is limited by antinutritional factors in the products. This study investigated the effect of different dietary levels of CSM and CSO supplementation on the laying performance and egg quality of laying hens. A total of 162 24‐week‐old Hy‐Line brown laying hens were randomly assigned to diets supplemented with 0, 6%, or 12% CSM and 0, 2%, or 4% CSO in a 3 × 3 factorial design. During the 8‐week feeding trial, laying performance and egg quality parameters were measured weekly. Furthermore, a texture profile analysis (TPA) of the egg yolks was conducted, and the fatty acid profiles and protein composition of the yolks were measured to further determine egg quality. CSM supplementation decreased (*p* < 0.01) egg production and feed efficiency and increased (*p* < 0.01) yolk color, eggshell rate, and shell thickness, but had no significant effects on the TPA parameters, fatty acid profiles, and protein components of egg yolks. CSO supplementation resulted in decreases (*p* < 0.01) in egg production, egg weight, and feed efficiency and an increase (*p* < 0.01) in yolk color. In addition, CSO supplementation with two weeks of cold storage changed the physical properties of boiled egg yolks, as indicated by increased (*p* < 0.01) hardness, springiness, cohesiveness, and chewiness. Furthermore, 4% CSO supplementation increased the ratio of saturated/monounsaturated fatty acids (SAFA/MUFA) and the protein content of egg yolks, which was accompanied by a modified protein composition. These results indicate that CSM supplementation reduces laying performance and egg quality, and CSO supplementation decreases laying performance and results in egg yolk hardening by modifying its components.

## 1 INTRODUCTION

In the poultry industry, soybean meal is one of the most commonly used ingredients because of its high level of protein and balanced amino acid profile, but its limited availability and relatively high price necessitate that different protein sources be studied. As an attractive alternative protein source, cottonseed meal (CSM) has been considered for use in poultry diets (Reid, Galavizmoreno, & Maiorino, 1987). Although its protein content and quality are lower than those of soybean meal, CSM is feasible for use in layer diets due to the lower energy and protein requirements of laying hens compared with those of broilers (Davis, Lordelo, & Dale, 2002). Additionally, cottonseed oil (CSO) is mainly composed of linoleic (52%), palmitic (24%), and oleic (22%) acids (O'Brien, Jones, King, Wakelyn, & Wan, 2005) and is rich in fat‐soluble vitamins, which can be used to supply essential fatty acids and additional nutrients. However, several antinutritional factors have been reported in CSM and CSO that limit their use.

Free gossypol (FG) in CSM is associated with depressed egg production (Gilani, Kermanshahi, Golian, & Tahmasbi, 2013), reduced feed efficiency (Yuan et al., 2014), and egg yolk discoloration (Davis et al., 2002) in layers, but He et al. (2015) proposed that these adverse effects may not be due to FG but the high level of arginine in low‐gossypol CSM diets. Low levels of available lysine also influence the use of CSM (Anderson & Warnick, 1966; Lyman, Chang, & Couch, 1953), but this could be corrected through synthetic lysine supplementation (Hassanabadi, Heidariniya, & Shahir, 2009; Watkins, Skinner, Adams, & Waldroup, 1993). Moreover, the type of fiber likely contributes to lower performance (Watkins et al., 1993).

The presence of cyclopropenoid fatty acids (CFA), which consist of malvalic and sterculic acids (Hendricks, Sinnhuber, Loveland, Pawlowski, & Nixon, 1980), in CSO has been reported to adversely affect production performance and inhibit the desaturation of saturated fatty acids (SAFA) (Raju & Reiser, 1967). Several feeding trials have been conducted to determine the effects of crude CSO in poultry diets (Aguiar et al., 2016; Bai, Chen, Guo, Ge, & Huang, 2014; Lima et al., 2016), and the results indicated that crude CSO could increase the color intensity and hardness of egg yolks. Since both FG and CFA are present in crude CSO, it is difficult to determine the main impact factor exerting these adverse effects.

Supersolidified egg yolks were found in hens consuming cottonseed and cottonseed products, but the main impact factor causing yolk hardening remains controversial because previous dietary resource studies considered more than one antinutritional factor. Hence, in the current study, nongossypol CSO was used instead of crude CSO to determine the effects caused by CFA, and CSM was used instead of cottonseed and cottonseed cake to determine the effect of FG. In addition, three levels of CSO and CSM addition as well as their combinations were used.

The objectives of this study were to evaluate the effects of dietary CSO and CSM supplementation at different concentrations on laying performance and egg quality and to clarify the main dietary factor causing the hardening and component modification of egg yolks.

## 2 MATERIALS AND METHODS

### 2.1 Animals and management

All animal procedures used in this study were approved by the Institutional Animal Care and Use Committee of Huazhong Agricultural University, China. A total of 162 20‐week‐old Hy‐Line brown hens were randomly assigned to 54 cages (three birds per cage) that provided 455 cm^2^ of space per hen. The birds had free access to water via a nipple drinker and were fed 120 g of diet per day with a manual feeder. After 4 weeks of prefeeding, the experimental diets were supplied for 8 weeks.

### 2.2 Dietary treatments

A total of nine treatments with three replications (two adjunct cages as one replicate) were administered in a 3 × 3 factorial design, and as shown in Table 1, three concentrations of both CSM (0, 6% and 12%) and CSO (0, 2% and 4%) were used. The CSM contained 0.8% oil and 693.81 mg/kg FG. To maintain similar energy intake and feed characteristics, 4% vegetable oil composed of different proportions of soybean oil (SBO) and non‐FG CSO was used for each experimental diet. The fatty acid profiles of SBO and CSO are presented in Table 1. The nutritional composition of all experimental diets was designed according to NRC (Dale, 1994) recommendations (Table 2).

**Table 1:** The fatty acid profiles (%) of dietary oils

Fatty acid^a^	Soybean oil	Cottonseed oil
c14:1∆^9^	0.02	0.01
C14:0	0.18	0.65
C16:1∆^9^	0.12	0.59
C16:0	11.96	20.37
C17:2∆^7, 12^	0.01	0.05
C17:1∆^10^	0.10	0.10
C17:0	0.10	0.07
C18:2*n*‐6	55.06	58.22
C18:1*n*‐9	25.42	15.81
C18:3*n*‐3	1.23	—
C18:0	4.39	2.68
C18:2 cla	0.63	0.39
Malvalic acid, C18:1 cpe	—	0.12
Sterculic acid, C19:1 cpe	—	0.06
Dihydrosterculic acids, C19:0 cpa	—	0.12
C20:2n‐6	0.04	0.08
C20:1n‐9	0.36	0.36
C20:0	0.39	0.32
SAFA	17.02	24.1
MUFA	25.66	16.5
PUFA	56.97	58.74
CFA	—	0.29

a cla, conjugated linoleic acid; cpe, cyclopropene; cpa, cyclopropane; SAFA, saturated fatty acid; PUFA, monounsaturated fatty acid; PUFA, polyunsaturated fatty acid; CFA, cyclopropenoid fatty acid (cpe + cpa).

**Table 2:** Composition of experimental diets^a^

Items	Treatments^a^								
	CON	O_0_M_6_	O_0_M_12_	O_2_M_0_	O_2_M_6_	O_2_M_12_	O_4_M_0_	O_4_M_6_	O_4_M_12_
Ingredient, %									
Corn (7.8% CP)	52.20	52.20	52.20	52.20	52.20	52.20	52.20	52.20	52.20
Wheat Bran (15.7% CP)	6.00	6.00	6.00	6.00	6.00	6.00	6.00	6.00	6.00
Soybean meal (44.2% CP)	26.10	20.10	14.10	26.10	20.10	14.10	26.10	20.10	14.10
Cottonseed meal (43.5% CP)	0.00	6.00	12.00	0.00	6.00	12.00	0.00	6.00	12.00
Cottonseed oil	0.00	0.00	0.00	2.00	2.00	2.00	4.00	4.00	4.00
Soybean oil	4.00	4.00	4.00	2.00	2.00	2.00	0.00	0.00	0.00
Dicalcium phosphate	1.53	1.38	1.22	1.53	1.38	1.22	1.53	1.38	1.22
Limestone	8.76	8.85	8.96	8.76	8.85	8.96	8.76	8.85	8.96
Salt	0.30	0.30	0.30	0.30	0.30	0.30	0.30	0.30	0.30
Lysine	0.00	0.06	0.11	0.00	0.06	0.11	0.00	0.06	0.11
Methionine	0.11	0.11	0.11	0.11	0.11	0.11	0.11	0.11	0.11
Premix	1.00	1.00	1.00	1.00	1.00	1.00	1.00	1.00	1.00
Total	100.00	100.00	100.00	100.00	100.00	100.00	100.00	100.00	100.00
Calculated nutrient level									
ME, MJ/kg	11.45	11.36	11.27	11.51	11.42	11.33	11.57	11.47	11.38
CP, g/kg	166.6	166.6	166.6	166.6	166.6	166.6	166.6	166.6	166.6
Ca, g/kg	35.06	35.01	35.02	35.06	35.01	35.02	35.06	35.01	35.02
Total P, g/kg	6.10	6.11	6.10	6.10	6.11	6.10	6.10	6.11	6.10
Available P, g/kg	3.84	3.69	3.51	3.84	3.69	3.51	3.84	3.69	3.51
Lysine, g/kg	8.54	8.59	8.55	8.54	8.59	8.55	8.54	8.59	8.55
Methionine, g/kg	3.49	3.48	3.48	3.49	3.48	3.48	3.49	3.48	3.48
Free gossypol, mg/kg	0.00	41.63	83.26	0.00	41.63	83.26	0.00	41.63	83.26
Cyclopropenoid fatty acid, mg/kg	0.0	0.0	0.0	58.0	58.0	58.0	116.0	116.0	116.0

a CON, control diet; O_0_M_6_, diet with 0% cottonseed oil and 6% cottonseed meal; O_0_M_12_, diet with 0% cottonseed oil and 12% cottonseed meal; O_2_M_0_, diet with 2% cottonseed oil and 0% cottonseed meal; O_2_M_6_, diet with 2% cottonseed oil and 6% cottonseed meal; O_2_M_12_, diet with 2% cottonseed oil and 12% cottonseed meal; O_4_M_0_, diet with 4% cottonseed oil and 0% cottonseed meal; O_4_M_6_, diet with 4% cottonseed oil and 6% cottonseed meal; O_4_M_12_, diet with 4% cottonseed oil and 12% cottonseed meal.

### 2.3 Laying performance and egg quality

For each replicate (*n* = 3) during the trial, hen‐day egg production, egg weight, egg mass, and abnormal (broken, soft shelled, and shell‐less) egg rate were recorded daily and averaged by week. Feed consumption was measured weekly, and feed efficiency was expressed as feed per egg and feed per gram of egg. A total of 864 eggs (4 eggs × 3 replicates × 9 treatments × 8 weeks) were collected for egg quality analysis. Egg weight, Haugh units (Haugh, 1937), and yolk color were automatically tested with an Egg Multitester (EMT‐7300; Touhoku Rhythm Co., Ltd), and shell strength was measured with an Egg Shell Force Gauge (EFG‐0503; Robotmation Co., LTD). The egg index was calculated as the ratio of egg length to egg width as measured by a Vernier caliper. The weights of the yolk, albumen, and eggshell were recorded and used to calculate the ratios of yolk, albumen, and eggshell to egg weight. Eggshell thickness was measured using a micrometer caliper and recorded as the average thickness of the blunt end, middle, and tip.

### 2.4 Texture profile analysis of boiled egg yolk

Twelve eggs were collected from each group at the end of the 4th, 6th, and 8th weeks, and these eggs were randomly divided into three equal groups. Group I was pretreated immediately after collection, and groups II and III were stored at 4°C or room temperature (25°C), respectively, for 14 days before pretreatment. The pretreatment procedure was as follows: The eggs were initially boiled for 10 min at 100°C and transferred into cold water for approximately 30 min. Then, all eggs were stored in a freezer at 4°C until the texture profile analysis (TPA), which was performed on the next day. Whole egg yolks were manually separated from egg white and prepared to be tested at room temperature. They were subjected to a double compression test (20% compression) using a texture profile analyzer (TA.XT Plus, Stable Micro Systems) fitted with a ±30 kg load cell. Measurements were performed using a 100‐mm compression plate with a test speed of 1 mm/s and a delay period of 5 s. TPA parameters were obtained using the Texture Exponent software package of the analyzer.

### 2.5 Fatty acid profile analysis of egg yolk

Six eggs were randomly collected from each replicate at the 6th experimental week. Egg yolks were manually separated (McBee & Cotterill, 1979) and blended using a magnetic stirrer (MS‐H‐Pro^+^; DLAB) at 4°C for 30 min. Then, 1 ml of the mixed yolk liquid was diluted with 5 ml of deionized water, and 4 ml of 10 mol/L hydrochloric acid was added. The reaction solution was placed at room temperature for 30 min followed by incubation at 100°C for 10 min. After cool, the solutions were stirred with a fourfold volume of a methanol‐chloroform mixture (methanol–chloroform volume ratio = 1:2) for 30 min. After centrifugation, 1 ml of the chloroform layer was isolated, and the residual solvent was removed using nitrogen flushing method.

Egg yolk oil was extracted and detected by gas chromatography‐mass spectrometry (GC‐MS) based on the procedure described by Liu et al. (2013) with minor modifications. The extracted lipid was dissolved in 950 μl of 5% sulfuric acid‐methanol solution. Pentadecanoic acid was added as an internal standard and then reacted at 90°C for 2 hr in a sealed vial. Next, 1 ml of hexane was used to extract the fatty acid methyl esters after the addition of 1 ml of 0.9% (w/v) sodium chloride solution. After centrifugation, the supernatant was filtered for GC‐MS analysis.

An Agilent 7890/5977 GC‐MS system equipped with the HP‐5ms column (30 m × 0.25 mm × 0.25 μm; Agilent Technologies) was used for GC‐MS analysis. One μl of sample was injected into the system, and the GC oven temperature was programmed from 180°C (2 min) to 250°C (1 min) at 5°C/min. The flow rate of the carrier gas was set at 1 ml/min, the split ratio was 1:20, the interface temperature was 250°C, and the ion‐source temperature was 230°C. Mass spectra were acquired under electron ionization mode at −70 eV using a scan ranging from 50 to 450 m/z with a solvent delay of 3 min. Peak identification was based on the National Institute of Standards and Technology (NIST14) database.

### 2.6 Protein content and composition analysis of egg yolk

Six eggs were randomly selected from each replicate, and the yolks were mixed by a homogenizer (T18 digital ULTRA‐TURRAX, IKA Co.). Protein concentrations were measured with a BCA protein assay kit (Beyotime), and the results were expressed as g proteins/ml egg yolk. An equal volume of 2 × loading buffer was added to the sample and heated in boiling water for 10 min, and the lysates were then centrifuged to obtain the supernatant. Proteins (approximately 25 μg) in the samples were separated by SDS‐PAGE, as described by Laemmli (1970). Band images were obtained using a ChemiDoc™ MP Imaging System (Bio‐Rad Laboratories), and band intensity was analyzed with Image Lab 5.1 software (Bio‐Rad).

### 2.7 Statistical analysis

All parameters were evaluated by multifactor analysis of variance (ANOVA) performed in R 3.3.3 (R Core & Team, 2017). The fitted factors for laying performance, egg quality parameters, fatty acid profiles, and protein composition were the levels of CSO (0, 2% or 4%) and CSM (0, 6% or 12%); the storage method factor (*n* = 3) was also included in the TPA. The CSO × CSM covariance was fitted for all traits, and the interactions between the storage methods and the feed composition (CSO and CSM) were tested for the TPA measurements. Multiple comparisons were performed using Tukey's honestly significant difference test, and *p* < 0.05 was used to indicate significance.

## 3 RESULTS

### 3.1 Laying performance

As shown in Table 3, laying performance was adversely affected by CSO consumption. Supplementation with 2% or 4% CSO significantly reduced egg weight, and 4% CSO significantly decreased hen‐day egg production and egg mass and increased feed per gram of egg (*p* < 0.05). Supplementation with 6% CSM increased egg weight (*p* < 0.05), feed per egg (*p* < 0.01) and feed per gram of eggs (*p* < 0.01), and reduced hen‐day egg production (*p* < 0.05). However, the high level (12%) of CSM intake increased hen‐day egg production (*p* < 0.05). The interaction effects on laying performance were observed between CSO and CSM.

**Table 3:** Effects of cottonseed oil (CSO) and cottonseed meal (CSM) supplementation on laying performance

Item	Hen‐day egg production, %	Egg weight, g	Egg mass, g/hen per day	Feed per egg, g	Feed per gram of egg, g/g	Abnormal egg rate, %
Treatment^a^						
CON	95.8^c^	58.5^cd^	56.6^bc^	111.7^b^	1.91^bcd^	0.63^ab^
O_0_M_6_	95.0^c^	59.5^d^	57.3^c^	112.9^b^	1.90^abc^	0.30^a^
O_0_M_12_	95.1^c^	58.4^cd^	56.5^bc^	114.6^b^	1.96^cd^	0.65^ab^
O_2_M_0_	92.0^bc^	58.5^cd^	55.5^bc^	102.4^a^	1.75^a^	0.34^a^
O_2_M_6_	94.7^c^	58.1^bc^	56.4^bc^	112.2^b^	1.93^bcd^	0.90^ab^
O_2_M_12_	95.3^c^	56.6^a^	54.6^bc^	112.8^b^	2.00^cde^	0.63^ab^
O_4_M_0_	88.5^b^	57.0^ab^	52.9^b^	118.0^bc^	2.08^de^	1.93^b^
O_4_M_6_	76.9^a^	58.6^cd^	49.8^a^	125.6^c^	2.15^e^	0.84^ab^
O_4_M_12_	95.7^c^	57.6^abc^	56.0^bc^	102.1^a^	1.77^ab^	0.62^ab^
*SEM*	0.01	0.11	0.33	0.81	0.01	0.11
*p*‐Value	<0.001	<0.001	<0.001	<0.001	<0.001	0.023
Levels of CSO, %						
0	95.3^b^	58.8^b^	56.8^b^	113.1^ab^	1.92^a^	0.52
2	94.0^b^	57.7^a^	54.5^b^	109.2^a^	1.89^a^	0.62
4	87.0^a^	57.7^a^	52.9^a^	115.2^b^	2.00^b^	1.13
Levels of CSM, %						
0	92.1^b^	58.0^a^	55.0	110.7^a^	1.91^a^	0.97
6	88.9^a^	58.7^b^	54.5	116.9^b^	1.99^b^	0.68
12	95.4^c^	57.5^a^	55.7	109.8^a^	1.91^ab^	0.62
Source of variation, *p*‐value						
CSO	<0.001	<0.001	<0.001	0.001	0.001	0.042
CSM	<0.001	<0.001	0.138	<0.001	0.008	0.362
CSO × CSM	<0.001	<0.001	<0.001	<0.001	<0.001	0.037

Note

^a–e^Means within a column marked with different superscripts differ significantly at *p* < 0.05.

Abbreviation: *SEM*, standard error of the mean.

a CON, control diet; O0M6, diet with 0% cottonseed oil and 6% cottonseed meal; O0M12, diet with 0% cottonseed oil and 12% cottonseed meal; O2M0, diet with 2% cottonseed oil and 0% cottonseed meal; O2M6, diet with 2% cottonseed oil and 6% cottonseed meal; O2M12, diet with 2% cottonseed oil and 12% cottonseed meal; O4M0, diet with 4% cottonseed oil and 0% cottonseed meal; O4M6, diet with 4% cottonseed oil and 6% cottonseed meal; O4M12, diet with 4% cottonseed oil and 12% cottonseed meal.

### 3.2 Egg quality

The egg quality parameters are shown in Table 4. CSO supplementation affected the egg shell ratio (*p* < 0.05), yolk color (*p* < 0.01), and shell thickness (*p* < 0.05), but it did not influence the other egg quality parameters. Particularly, 2% CSO supplementation deepened (*p* < 0.05) yolk color. With increased dietary CSM supplementation, yolk color increased (*p* < 0.05). Furthermore, hens fed 12% CSM had a higher eggshell ratio (*p* < 0.05) and shell thickness (*p* < 0.05). There was a CSO × CSM interaction effect (*p* < 0.05) on the egg yolk ratio, yolk color, shell thickness, and egg index.

**Table 4:** Effects of cottonseed oil (CSO) and cottonseed meal (CSM) supplementation on egg quality

Item	Ratio of, %			Yolk color	Hungh unit	Shell strength, *N*	Shell thickness, mm	Egg index
	Yolk	Albumen	Shell					
Treatment^a^								
CON	24.3^ab^	64.3	12.0^ab^	3.83^a^	91.1	32.8	0.342^ab^	1.27
O_0_M_6_	24.2^ab^	64.1	12.1^ab^	4.20^b^	93.2	33.9	0.348^ab^	1.28
O_0_M_12_	23.9^a^	63.7	12.4^b^	4.46^b^	93.2	32.3	0.357^b^	1.29
O_2_M_0_	24.4^ab^	63.8	12.2^ab^	4.05^ab^	93.9	32.4	0.350^ab^	1.29
O_2_M_6_	23.7^a^	64.9	11.9^a^	4.18^ab^	93.6	31.2	0.336^a^	1.28
O_2_M_12_	23.9^ab^	63.8	12.4^ab^	4.92^c^	91.5	33.1	0.350^ab^	1.29
O_4_M_0_	24.1^ab^	63.6	12.3^ab^	4.20^ab^	92.6	32.3	0.347^ab^	1.29
O_4_M_6_	23.4^a^	64.4	12.2^ab^	4.23^b^	93.0	33.0	0.351^ab^	1.28
O_4_M_12_	25.1^b^	63.1	12.5^b^	4.43^b^	89.3	34.7	0.355^b^	1.28
*SEM*	0.11	0.30	0.02	0.03	0.20	0.23	0.00	0.00
*p*‐Value	<0.001	0.143	0.004	<0.001	0.090	0.182	0.002	0.017
Levels of CSO, %								
0	24.1	64.0	12.2	4.16^a^	92.5	33.0	0.349	1.28
2	24.0	64.0	12.1	4.39^b^	93.0	32.2	0.345	1.29
4	24.2	63.6	12.3	4.29^ab^	91.6	33.3	0.351	1.28
Levels of CSM, %								
0	24.3^ab^	63.9^ab^	12.2^a^	4.03^a^	92.5	32.5	0.346^a^	1.29
6	23.8^a^	64.3^b^	12.1^a^	4.20^b^	93.3	32.7	0.345^a^	1.28
12	24.3^b^	63.5^a^	12.4^b^	4.60^c^	91.3	33.3	0.354^b^	1.29
Source of variation, *p*‐value								
CSO	0.522	0.393	0.031	0.001	0.320	0.202	0.044	0.140
CSM	0.022	0.025	<0.001	<0.001	0.094	0.387	<0.001	0.064
CSO × CSM	0.002	0.459	0.278	<0.001	0.118	0.105	0.003	0.048

Note

^a‐c^Means within a column marked with different superscripts differ significantly at *p* < 0.05.

Abbreviation: *SEM*, standard error of the mean.

a CON, control diet; O_0_M_6_, diet with 0% cottonseed oil and 6% cottonseed meal; O_0_M_12_, diet with 0% cottonseed oil and 12% cottonseed meal; O_2_M_0_, diet with 2% cottonseed oil and 0% cottonseed meal; O_2_M_6_, diet with 2% cottonseed oil and 6% cottonseed meal; O_2_M_12_, diet with 2% cottonseed oil and 12% cottonseed meal; O_4_M_0_, diet with 4% cottonseed oil and 0% cottonseed meal; O_4_M_6_, diet with 4% cottonseed oil and 6% cottonseed meal; O_4_M_12_, diet with 4% cottonseed oil and 12% cottonseed meal.

### 3.3 Texture profile analysis of boiled egg yolk

**Figure 1:**
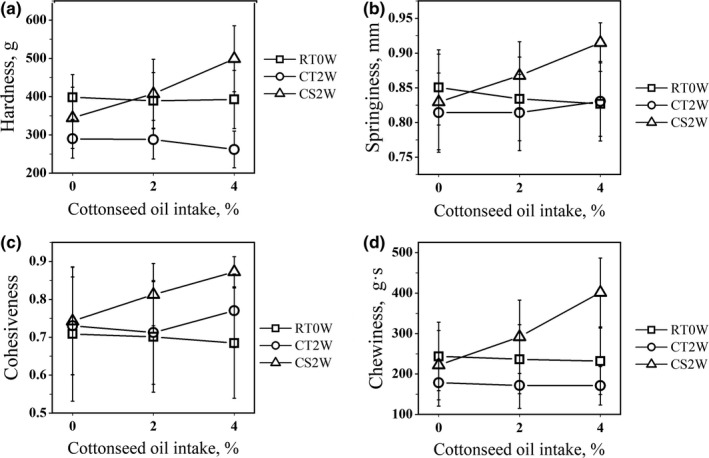
Texture profile analysis of boiled egg through different storage methods and produced by birds with different levels of cottonseed oil (CSO) supplementation. RT0W = room temperature stored 0 weeks; RT2W = room temperature stored 2 weeks; CS2W = cold stored 2 weeks. The TPAs of egg that produced by layers feed 0% CSO are similar in different storage method. Egg yolk TPAs are not changed significantly within 2 weeks at room temperature. However, the TPAs of eggs stored at 4°C for two weeks are positively correlated with the percentage of CSO intake

The texture profiles of boiled egg yolks are presented in Table 5. CSM supplementation did not affect TPA parameters, but with increasing CSO supplementation, the hardness, springiness, cohesiveness, resilience, and chewiness of egg yolk increased (*p* < 0.001) while adhesiveness decreased. Additionally, the storage condition of eggs significantly affected egg yolk TPA parameters. Two weeks of storage at room temperature decreased the hardness and chewiness of egg yolk, but cold storage increased springiness, cohesiveness, resilience, and chewiness (*p* < 0.05). Furthermore, an interaction was observed between CSO and storage method (*p* < 0.01), and the synergistic effects are shown in Figure 1. CSO supplementation did not significantly affect TPA parameters when eggs were tested immediately or stored at room temperature, but the hardness, springiness, cohesiveness, and chewiness of the yolks kept in a refrigerated cabinet were positively correlated (*p* < 0.05) with the level of CSO consumption.

**Table 5:** Effects of cottonseed oil (CSO) and cottonseed meal (CSM) supplementation on egg yolk texture properties^a^

Item	HARD, g	ADHE, g·s	SPRI, mm	COHE	RESI	CHEW, g·s
Treatments^b^						
CON	338.3^ab^	1.184	0.808^ab^	0.706^a^	0.417^a^	207.0^a^
O_0_M_6_	336.8^ab^	1.234	0.797^a^	0.708^ab^	0.420^a^	201.7^a^
O_0_M_12_	335.6^a^	1.183	0.810^ab^	0.721^abc^	0.417^a^	206.0^a^
O_2_M_0_	360.5^abc^	1.086	0.821^ab^	0.727^abc^	0.438^ab^	228.4^ab^
O_2_M_6_	367.3^abc^	1.091	0.839^ab^	0.754^abc^	0.448^ab^	241.3^abc^
O_2_M_12_	379.3^bc^	1.076	0.838^ab^	0.772^bc^	0.466^b^	258.4^bc^
O_4_M_0_	399.6^c^	1.041	0.846^b^	0.768^c^	0.475^b^	276.7^c^
O_4_M_6_	403.1^c^	0.988	0.842^ab^	0.752^bc^	0.469^b^	269.8^bc^
O_4_M_12_	371.1^abc^	1.095	0.831^ab^	0.774^c^	0.468^b^	250.0^bc^
*SEM*	3.576	0.022	0.004	0.005	0.003	3.541
*p*‐Value	<0.001	0.326	0.005	0.001	<0.001	<0.001
Levels of CSO, %						
0	336.9^a^	1.201^b^	0.805^a^	0.712^a^	0.418^a^	204.9^a^
2	369.0^b^	1.084^ab^	0.833^b^	0.751^b^	0.450^b^	242.6^b^
4	391.0^c^	1.042^a^	0.840^b^	0.764^b^	0.471^c^	265.3^c^
Levels of CSM, %						
0	365.5	1.104	0.825	0.733	0.443	236.9
6	368.8	1.106	0.826	0.738	0.445	237.4
12	361.9	1.118	0.826	0.756	0.450	238.0
Storage method^c^						
RT0W	391.4^b^	1.137^ab^	0.807^a^	0.673^a^	0.400^a^	228.2^b^
RT2W	281.0^a^	1.181^b^	0.806^a^	0.736^b^	0.414^a^	171.6^a^
CS2W	428.3^b^	1.016^a^	0.860^b^	0.805^c^	0.516^b^	310.6^c^
Source of variances, *p*‐value						
CSO	<0.001	0.019	<0.001	<0.001	<0.001	<0.001
CSM	0.487	0.966	0.980	0.099	0.466	0.982
Storage	<0.001	0.014	<0.001	<0.001	<0.001	<0.001
CSO × CSM	0.003	0.830	0.410	0.540	0.185	0.003
CSO × Storage	<0.001	0.002	<0.001	<0.001	<0.001	<0.001
CSM × Storage	0.846	0.364	0.357	0.597	0.608	0.993

Note

^a‐c^Means within a column marked with different superscripts differ significantly at *p* < 0.05.

Abbreviation: *SEM*, standard error of the mean.

a ADHE, adhesiveness; CHEW, chewiness; COHE, cohesiveness; HARD, hardness; RESI, resilience; SPRI, springiness.

b CON, control diet; O_0_M_6_, diet with 0% cottonseed oil and 6% cottonseed meal; O_0_M_12_, diet with 0% cottonseed oil and 12% cottonseed meal; O_2_M_0_, diet with 2% cottonseed oil and 0% cottonseed meal; O_2_M_6_, diet with 2% cottonseed oil and 6% cottonseed meal; O_2_M_12_, diet with 2% cottonseed oil and 12% cottonseed meal; O_4_M_0_, diet with 4% cottonseed oil and 0% cottonseed meal; O_4_M_6_, diet with 4% cottonseed oil and 6% cottonseed meal; O_4_M_12_, diet with 4% cottonseed oil and 12% cottonseed meal.

c RT0W, room temperature stored 0 weeks; RT2W, room temperature stored 2 weeks; CS2W, cold stored 2 weeks.

### 3.4 Egg yolk fatty acid profile

As shown in Table 6, no significant difference in fatty acid profiles was observed between egg yolks under 12% CSM supplementation and the control group. CSO supplementation resulted in decreases in C16:1 (*p* < 0.01) and C17:1 (*p* < 0.05), C18:1 (*p* < 0.01) as well as increases in C16:0 (*p* < 0.05), C17:0 (*p* < 0.01), and C18:0 (*p* < 0.01). Thus, a significant increase in total SAFA content and a decrease in total monounsaturated fatty acid (MUFA) content were observed due to 4% CSO supplementation. Furthermore, CSO supplementation increased the contents of C20:4n‐6 (*p* < 0.05), C22:5n‐6 (*p* < 0.01), polyunsaturated fatty acids (PUFA) (*p* < 0.05) and Σn‐6 (*p* < 0.01), and decreased (*p* < 0.01) docosahexaenoic acid (DHA) and the n‐3/n‐6 ratio. However, the CFA in CSO was not detected in the yolks. No interaction effect between CSO and CSM was observed on fatty acid composition.

**Table 6:** Effects of cottonseed oil (CSO) and cottonseed meal (CSM) supplementation on egg yolk fatty acid profiles (%)

Fatty acid^b^	Treatments^a^				*SEM*	*p*‐Value		
	CON	O_0_M_12_	O_4_M_0_	O_4_M_12_		CSO	CSM	CSO × CSM
C14:1∆^9^	0.35	0.37	0.41	0.28	0.05	0.882	0.664	0.562
C14:0	0.67	0.77	0.93	0.76	0.09	0.527	0.849	0.496
C16:1	0.59^b^	0.48^ab^	0.15^a^	0.17^a^	0.07	0.001	0.563	0.445
C16:1∆^9^	1.55^a^	1.49^a^	0.44^b^	0.45^b^	0.17	<0.001	0.885	0.794
C16:0	24.89	26.00	29.09	27.80	0.67	0.023	0.937	0.293
C17:2∆^7,12^	0.17	0.12	0.21	0.11	0.03	0.912	0.357	0.736
C17:1∆^10^	0.13	0.12	0.08	0.07	0.01	0.038	0.649	0.962
C17:0	0.16^ab^	0.15^a^	0.24^bc^	0.21^b^	0.01	0.001	0.215	0.607
C18:3*n*‐6	0.15	0.17	0.12	0.10	0.02	0.167	0.928	0.665
C18:2*n*‐6	18.39	18.68	21.25	25.17	1.07	0.020	0.229	0.295
C18:1*n*‐9c	31.33^b^	29.18^b^	17.03^a^	18.40^a^	1.98	<0.001	0.765	0.200
C18:1*n*‐9t	3.90^b^	3.20^ab^	2.09^ab^	1.42^a^	0.36	0.006	0.192	0.975
C18:0	12.49^a^	13.41^a^	22.04^b^	19.72^b^	1.32	<0.001	0.560	0.199
C20:4*n*‐6(ARA)	3.08	3.15	2.74	2.59	0.10	0.030	0.803	0.537
C20:3*n*‐6	0.19	0.21	0.28	0.24	0.02	0.140	0.715	0.455
C20:2*n*‐6	0.32	0.36	0.60	0.42	0.05	0.055	0.416	0.186
C20:1*n*‐9	0.23	0.26	0.43	0.22	0.04	0.238	0.210	0.108
C20:0	0.28	0.36	0.37	0.14	0.05	0.563	0.501	0.201
C22:5*n*‐6	0.17^a^	0.32^a^	0.77^b^	0.85^b^	0.10	<0.001	0.289	0.738
C22:6*n*‐3(DHA)	0.78	1.04	0.46	0.64	0.09	0.037	0.162	0.779
C22:4*n*‐6	0.17	0.17	0.27	0.24	0.02	0.039	0.697	0.652
ΣSAFA	38.49^a^	40.68^ab^	52.67^c^	48.63^bc^	1.98	0.001	0.687	0.198
ΣMUFA	38.07^b^	35.09^b^	20.62^a^	21.02^a^	2.45	<0.001	0.290	0.176
ΣPUFA	23.43	24.23	26.71	30.35	1.04	0.015	0.183	0.376
Σ*n*‐3	0.78	1.04	0.46	0.64	0.09	0.037	0.162	0.780
Σ*n*‐6	22.48^a^	23.06^ab^	26.04^ab^	29.60^b^	1.05	0.008	0.191	0.333
*n*‐3/*n*‐6	0.03^ab^	0.05^b^	0.02^a^	0.02^a^	0.00	0.003	0.129	0.476

Note

^a‐c^Means within a row marked with different superscripts differ significantly at *p* < 0.05.

a CON, control diet; O_0_M_12_, diet with 0% cottonseed oil and 12% cottonseed meal; O_4_M_0_, diet with 4% cottonseed oil and 0% cottonseed meal; O_4_M_12_, diet with 4% cottonseed oil and 12% cottonseed meal.

b ARA, arachidonic acid; DHA, docosahexaenoic acid; MUFA, monounsaturated fatty acid; PUFA, polyunsaturated fatty acid; SAFA, saturated fatty acid.

### 3.5 Egg yolk protein content and composition

As shown in Table 7, CSM supplementation decreased the concentration of apovitellenin II (*p* < 0.05) but did not affect the other proteins. However, the egg yolk protein content and composition were modified by CSO supplementation. The eggs of hens fed 4% CSO exhibited enhanced total protein content (*p* < 0.05) and increased concentration of apovitellin 1 + 2 (*p* < 0.05), apovitellin 3 (*p* < 0.01), apovitellenin Va (*p* < 0.01), apovitellenin V/ovotransferrin (*p* < 0.01), apovitellenin IV/α‐livetin (*p* < 0.01), and β‐livetin (*p* < 0.05). In addition, CSO supplementation decreased the yolk concentrations of apovitellenin I (*p* < 0.05) and apovitellenin II (*p* < 0.01). Furthermore, there was an interaction effect between CSM and CSO on the concentrations of apovitellenin Va (*p* < 0.05), apovitellin 5 + 6 (*p* < 0.01), apovitellenin IV/α‐livetin (*p* < 0.01), and ovalbumin (*p* < 0.05).

**Table 7:** Effects of cottonseed oil (CSO) and cottonseed meal (CSM) supplementation on content and composition of egg yolk protein (%)

Est‐MW, kDa	Ture‐MW, kDa	Identified name	Treatments^a^				*SEM*	*p*‐Value		
			CON	O_0_M_12_	O_4_M_0_	O_4_M_12_		CSO	CSM	CSO × CSM
233	211	Apovitellenin Via^b^	1.88	2.04	2.04	1.58	0.10	0.462	0.457	0.144
213	203	γ‐Livetin/apovitellenin VI^b^	11.96	11.89	12.47	12.02	0.17	0.407	0.489	0.609
163	140	Apovitellin 1+2^c^	0.63	0.55	0.67	0.79	0.03	0.029	0.713	0.100
151	122	Apovitellenin Va^b^	1.31^a^	1.12^a^	1.38^ab^	1.83^b^	0.09	0.006	0.262	0.017
117	110	Apovitellin 3^c^	14.96^a^	15.20 ^a^	16.75^b^	16.49^b^	0.26	0.001	0.976	0.374
108	105	Apovitellin 4^c^	4.78	4.59	4.10	5.18	0.18	0.888	0.215	0.092
91	90	Apovitellenin V^b^/ovotransferrin	2.39^a^	2.44^a^	2.75^a^	3.51^b^	0.15	0.002	0.034	0.054
82	78	Apovitellin 5+6^c^	5.60^ab^	5.34^a^	5.54^a^	6.01^b^	0.08	0.016	0.318	0.007
69	75/70	Apovitellenin IV^b^/α‐livetin	5.50^a^	6.43^b^	6.57^b^	6.50^b^	0.16	0.023	0.070	0.040
63	60	Apovitellenin IIIa^b^	2.71	2.85	3.02	2.91	0.05	0.087	0.875	0.217
57	55	Apovitellenin III^b^	4.35	4.57	4.47	4.22	0.10	0.601	0.930	0.320
47	50	apovitellin 7^c^	4.37	4.29	4.51	4.79	0.14	0.305	0.742	0.560
42	45	Ovalbumin	5.61^ab^	5.30^ab^	4.74^a^	6.15^b^	0.20	0.959	0.107	0.023
40	45	β‐Livetin	6.92	7.34	7.67	7.40	0.12	0.105	0.747	0.154
35	35/31	phosvitin/Apovitellin 8^c^	9.56	9.95	9.61	8.98	0.16	0.152	0.695	0.118
30	28	IgY‐light chain	3.79^b^	3.93^b^	2.38^b^	0.53^a^	0.45	0.001	0.084	0.051
23	20	Apovitellenin II^b^	2.31^b^	2.17^b^	2.11^b^	1.78^a^	0.07	0.003	0.011	0.237
14	18	Apovitellenin I^b^	10.73^b^	9.58^ab^	8.71^a^	8.90^ab^	0.30	0.014	0.305	0.161
11	9	Apovitellenin Ia^b^	0.65	0.43	0.53	0.46	0.05	0.632	0.193	0.487
The total protein content, g/ml			16.95^a^	17.19^ab^	19.28^b^	19.39^b^	0.44	0.012	0.813	0.930

Note

^a,b^Means within a row marked with different superscripts differ significantly at *p* < 0.05.

a CON, control diet; O_0_M_12_, diet with 0% cottonseed oil and 12% cottonseed meal; O_4_M_0_, diet with 4% cottonseed oil and 0% cottonseed meal; O_4_M_12_, diet with 4% cottonseed oil and 12% cottonseed meal.

b Means name identified according to Burley and Sleigh (1980).

c Means name identified according to Kurisaki, Yamauchi, Isshiki, and Ogiwara (1981).

## 4 DISCUSSION

The results of this study confirmed that 4% CSO supplementation in a layer diet adversely impacted egg production and feed efficiency. In contrast, Aguiar et al. (2016) and Abdalqadir, Mohammed, Mohammad, Mohammad, and Arabi (2014) reported that the feed conversion ratio (FCR) was not affected by CSO intake in broilers. The significant effect of CSM supplementation on laying performance is similar to previous results indicating that high levels of dietary CSM significantly increased the feed gain ratio (Zeng et al., 2014) and decreased egg weights (Davis et al., 2002) (He et al., 2015) and production (Panigrahi, Plumb, & Machin, 1989), but He et al. (2015) and Adeyemo and Longe (2008) reported that low levels of CSM (5.00%, 9.83%, 14.42%, and 18.90%) and cottonseed cake (3.23%–12.97%) had no effect on egg production rates or feed efficiency.

Except for egg yolk discoloration, no significant adverse effects on egg quality were observed due to 4% CSO consumption, but more significant increases in yolk color due to 2%, 4%, or 6% CSO consumption were reported by Hamilton and Parkhurst (1990). Previous studies have proposed an enhancing effect of the interaction between CFA and FG intake on egg yolk discoloration (Kemmerer, Heywang, Nordby, & Phelps, 1962; Kemmerer, Heywang, & Vavich, 1960), so the reduced discoloration observed after CSO intake was probably due to the absence of FG. The current results indicate that using CSM as the protein source increased yolk color and eggshell thickness, which is consistent with the results of several previous studies that CSM supplementation discolored egg yolks to pink or brown (Davis et al., 2002; Heywang, Bird, & Altschul, 1955; Reid et al., 1987; Ryan, Kratzer, Grau, & Vohra, 1986). However, low concentrations of FG or CSM supplementation have not been reported to adversely affect yolk discoloration (Davis et al., 2002; Gilani et al., 2013; Ryan et al., 1986). Decreases in the Haugh unit due to CSM consumption was observed in previous studies (He et al., 2015; Yuan et al., 2014), but the nonsignificant effects of CSM consumption on shell strength, egg index, and the Haugh unit observed in this study are similar to some previous research (Adeyemo & Longe, 2008; Gilani et al., 2013; Yuan et al., 2014).

The physical properties of the egg yolk are major factors affecting consumer acceptance of fresh egg and yolk products, such as mayonnaise and baked cake. In the current study, the hardness, springiness, cohesiveness, resilience, and chewiness of boiled yolks increased with increasing CSO levels after 2 weeks of cold storage. Similarly, Bai et al. (2014) observed an increase in the TPA parameters (hardness, springiness, and cohesiveness) of boiled eggs due to crude CSO consumption, suggesting that FG and CFA are potential antinutritional factors. Qi et al. (2017) reported that 6% CSM supplementation with 491 mg/kg FG increased the hardness and elasticity of boiled egg yolks after cold storage, but Liu et al. (2016) reported that the consumption of 400 mg/kg FG did not significantly affect yolk springiness, which is consistent with our results. Thus, CFA supplementation from CSO may be the dietary factor that produces egg yolk hardening. Miller and Winter (1951) reported that mayonnaise made from frozen yolks was much stiffer than that made from fresh yolks, which indicates a similar effect of cold storage on yolk structure. Hence, CSO supplementation and cold storage should be avoided.

Heating, the protein contents and components, their types of bonds, and their combination with lipids have been reported to be crucial factors affecting egg yolk denaturation and hardening (Paraskevopoulou & Kiosseoglou, 1997; Tunick, Mackey, Smith, & Holsinger, 1991; Woodward & Cotterill, 1987); therefore, the fatty acid and protein composition were measured in this study. In this study, CSM supplementation had no significant effect on egg yolk components, which confirmed that no more than 83.26 mg/kg FG intake did not significantly impact egg yolk composition or structure. In contrast, CSM supplementation adversely affected the crude protein of egg yolk (Qi et al., 2017).

Cottonseed oil supplementation increased the SAFA concentration of egg yolks at the expense of MUFA, and Evans, Davidson, and Bandemer (1961) similarly observed that supplementation with crude CSO and *Sterculia foetida* seeds increased the stearic acid (18:0) and decreased the palmitoleic (16:1) acid and oleic acid (18:1) contents in egg yolk. These additives contain CFA, which was reported to irreversibly inhibit the activity of desaturases in rats (Raju & Reiser, 1967) and hens (Allen, Johnson, Fogerty, Pearson, & Shenstone, 1967) by acting on sulfhydryl enzyme groups. More recent research has suggested that the olefinic cyclopropenoid carbon at C9/C10 was the effective inhibitor (Fogerty, Johnson, & Pearson, 1972) that noncovalently bonds with desaturase (Pande & Mead, 1970). Therefore, the CFA in CSO is probably the key factor in lipid metabolism disorders. Furthermore, the significantly higher ratio of SAFA/MUFA supplied by CSO might exacerbate the poor fatty acid composition of egg yolks, whereas the additional SAFA supplied by tallow intake was not reported to increase the SAFA/MUFA ratio (Evans, Davidson, Larue, & Bandemer, 1963).

In addition, CSO consumption increased C22:4n‐6 and C22:5n‐6 and led to an increase of Σn‐6 with a decrease of n‐3/n‐6, which might have been caused by the higher linoleic acid (C18:2n‐6) intake, and modifications of fatty acid profiles could change the structure and physical properties of egg yolk. It was previously reported that an enhanced concentration of stearic acid could increase the density of lipoprotein and cause the lipoprotein to be transformed from VLDL into low‐density lipoproteins (LDL; Evans, Flegal, Foerder, Bauer, & Lavigne, 1977), which may lead to egg yolk hardening. Since research on human health has indicated that excess SAFA might increase the risk of cardiovascular disease (Souza et al., 2015), the nutritional value of eggs needs to be determined further.

An increase in protein content, which was reported to be positively correlated with the elastic modulus of an egg yolk solution during heating (Woodward & Cotterill, 1987), was observed when hens were fed 4% CSO. Egg yolk is composed of granules and plasma that mainly contain 70% high‐density lipoproteins (HDL) and 85% LDL, respectively. In this study, we observed modifications of the protein composition of egg yolks, including increases in lipovitellin 1, 2, 3 and changes in several plasma proteins (apovitellenin I, II, IV, V; ovotransferrin; and β‐livetin). Protein composition has been reported to play a crucial role in the physical properties of yolk gelation (Tunick et al., 1991), so the effect of the interaction between the increase in protein content and the modification of protein composition caused by CSO supplementation probably contributed to egg yolk hardening. Since the accuracy of the protein separation and abundance test performed by SDS‐PAGE is low, a high‐precision protein measurement, such as mass spectrometry, could improve the result. In addition, determining the molecular structure and connections in egg yolk gel will help explain the mechanism underlying egg yolk hardening.

## 5 CONCLUSIONS

In conclusion, CSM supplementation reduces the laying performance and egg quality of laying hens but has no significant effect on the physical properties and components of egg yolk. CSO supplementation reduces egg production and feed efficiency and results in egg yolk hardening when combined with refrigeration. In addition, supplementation with 4% non‐FG CSO increases the SAFA/MUFA ratio, increases the protein content, and modifies the protein composition of egg yolk, which caused egg yolk hardening during gelation.

## CONFLICT OF INTEREST

The authors declare that they do not have any conflict of interest.

## ETHICAL APPROVAL

The experimental animal procedure was approved by the Scientific Ethics Committee of Huazhong Agricultural University.
